# Enabling scale-up of mesoporous silicon for lithium-ion batteries: a systematic study of a thermal moderator

**DOI:** 10.1039/d0ra09000j

**Published:** 2021-01-19

**Authors:** Jake E. Entwistle, Siddharth V. Patwardhan

**Affiliations:** Department of Chemical and Biological Engineering, The University of Sheffield Mappin Street Sheffield S1 3JD UK s.patwardhan@sheffield.ac.uk

## Abstract

The volume expansion of silicon during cycling of a lithium-ion battery (LIB) leads to degradation and capacity loss. Porous silicon can address many of the issues faced by silicon active materials and has previously been shown to have excellent cyclability. Recently we have uncovered the mechanisms underpinning the pore evolution in magnesiothermic reduction (MgTR) of silica and further demonstrated that it has the potential to produce porous silicon in a scalable and economic manner [*J. Mater. Chem. A*, 2020, **8**, 4938]. However, the scalability of MgTR is affected by the large excess heat produced during reaction. Although previous studies have shown that NaCl can be used as a thermal moderator to mitigate this issue, this has not been systematically investigated, leading to a lack of knowledge on scalability of MgTR. Here, by carefully investigating the roles of NaCl, we show that the NaCl ratio and reduction temperature are the critical factors for controlling scale-up and the product properties. We identified the upper temperature limit of NaCl as a thermal moderator. Further, we systematically showed how the amount of NaCl and the reduction temperature affect the porous properties of the product silicon. Our results have established pathways for scaling-up this method such that it can now be taken forward to target specific porous silicon properties.

## Abstract 2

By identifying the critical controlling factors, this manuscript provides the first report enabling scale-up of mesoporous silicon, which is non-trivial due to the large excess heat produced.

## Graphical abstract

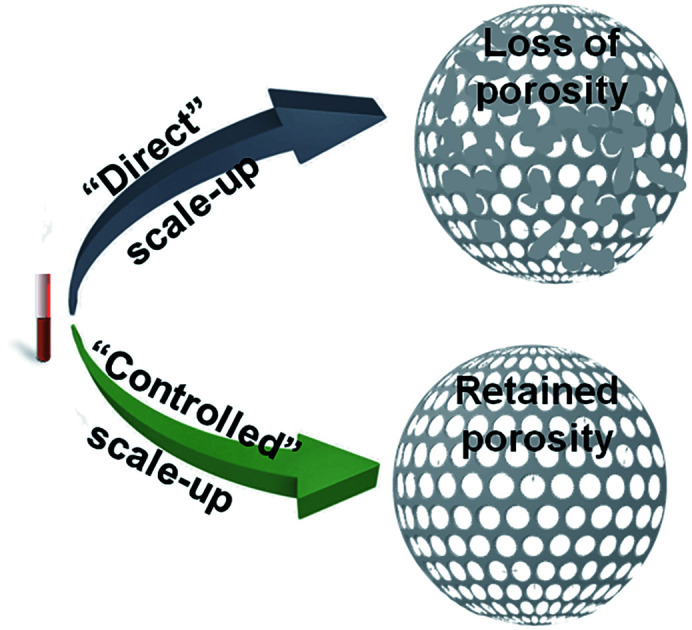

## 1. Introduction

The advent of the lithium-ion battery (LIB) revolutionised portable electronics and has a key role to play in the integration of large-scale renewable power and the electrification of transport. Increased energy and power density, along with lower cost of active materials will accelerate the adoption of LIBs in the above applications.^1^

The current commercial LIB uses an intercalating graphite anode where lithium-ions reversibly transfer within the planes of the graphite sheets resting in an interstitial site shared between six carbon atoms (C_6_Li), corresponding to a 371 mA h g^−1^ gravimetric and 830 mA h ml^−1^ volumetric capacity. Silicon has a much higher gravimetric and volumetric capacity than graphite at 3580 mA h g^−1^ and 2190 mA h ml^−1^. However the larger number of lithium-ions alloyed with silicon causes a 280% volume change during cycling.^2^ The large volume change of silicon presents a number of challenges; pulverisation of the silicon particles from expansion stresses, dislocation and electrical isolation of active material within the electrode, and continual solid electrolyte interface formation. These challenges lead to unstable capacity for silicon anodes and significant capacity fade during cycling as reported in our recent review.^3^ Porous silicon morphologies can address the issues related to volume expansion during lithiation. It has been shown that higher void fractions lead to lower induced stresses and larger pores have lower hoop stresses than smaller pores.^4,5^ Porous silicon can expand into its own pore volume, thereby limiting stresses on the material.^6^

For silicon material to make a positive impact into the LIB market by increasing cell capacity, it must be technically and economically feasible to synthesize on a large scale. Many synthesis routes to porous silicon require intricate chemical reactions, large energy inputs and specific reaction vessels.^3^ MgTR is promising due to its ability to provide a bulk synthesis route to mesoporous silicon. In our previous work,^7^ we have shown that the MgTR products of bioinspired silica (BIS) have excellent properties for LIB anode applications, in addition to improved economics from the use of BIS. In that work we also uncovered how silicon purities and crystallinities govern the pore properties of the reduced silicon.^7^ The wider applicability of that mechanism was validated by converting a range of silica precursors into mesoporous silicon of desired properties. However, the exothermic nature of the MgTR is known to cause issues when scaling-up this synthesis to commercial scales.^3^ Although this issue has been overcome by using slow heating rates and smaller batch sizes,^8^ such remedies make the process even less attractive for scale-up due to longer processing times and the need for multiple batches.

Unreactive thermal moderators such as sodium chloride can be used to limit the effects of self-heating in MgTR.^9,10^ As sodium chloride (NaCl) melts at 801 °C, associated with a large endothermic latent heat energy, it can moderate the MgTR temperature.^11^ Batchelor *et al.* showed that NaCl was critical for increasing batch size and managed to perform the reaction on a 30 g reaction scale.^12^ In addition to this, Luo *et al.* showed that when using NaCl thermal moderator, the initial silica precursor structure could be maintained in the reduced form.^11^ The addition of NaCl has also been reported to reduce the presence of Mg_2_Si in the product silicon.^13^ Although it has been previously shown that the reduction temperature is a key governing factor in pore properties of the silicon product,^7^ when using a thermal moderators, previous studies have not studied the effects of reduction temperature on the product quality. Specifically, the effect of the use of thermal moderators on the pore properties of silicon produced by MgTR has not been reported. This is critically important because the pore properties of silicon ultimately control its performance. Further, the effect of the amount of NaCl used on silicon product properties has been unexplored, let alone in conjunction with the effects of reduction temperature despite their importance in enabling scale-up. The lack of such information creates uncertainties in designing reliable and predictable scale-up of MgTR process. In this work, we explore the scale up of MgTR of BIS in conjunction with the use of thermal moderators. We hypothesise that silicon crystallite size may be strongly dependent on the upper temperature of reduction and that this will govern the pore properties of the product. We expect that the presence of NaCl will affect the crystallite formation, and hence we aim to investigate the amount of the NaCl used. The effect of MgTR process parameters and scales on the resulting materials performance as LIB anode materials will be studied in order to enable scale-up in the future.

## 2. Experimental

BIS was synthesised as described previously.^7^ Briefly, sodium metasilicate and pentaethylene hexamine (PEHA) were mixed inside of a 5 L Radley's lab reactor, followed by addition of 1 M hydrochloric acid (HCl) quickly in order for the pH to come to rest at 7.00 ± 0.05 within 2 minutes. After 5 minutes of reaction time, the product solution was collected, filtered and washed with deionised water before drying. BIS was calcined at 550 °C under air for 5 hours to remove all organic additives. The resulting calcined silica was ball milled for 5 minutes to give a fine homogeneous powder. Other silicas used were sourced commercially as follows. Precipitated silica refers to Perkasil KS4080PD and porous silica refers to microporous Syloid AL-1FP, both were kindly supplied by Grace. Silica Gel refers to ‘mesoporous silica gel 13 nm’ and quartz as ‘quartz white sand >99.995%’, both were purchased from Sigma.

The silica reduction was performed exactly as previously reported,^7^ except when appropriate mixing of the powders was followed by the addition of NaCl with the desired ratio, followed by a further thorough mixing with mortar and pestle. The amount of NaCl used herein is reported as a ratio of weights of (Mg + SiO_2_) and NaCl. Electrode and cell preparation, electrochemical performance measurements and materials characterisation using scanning electron microscopy, gas adsorption, X-ray diffraction and thermogravimetric analysis were all exactly as previously reported.^7^

## 3. Results and discussion

### 3.1 Scale-up of MgTR of BIS without any thermal moderator

The large exothermic nature of the MgTR reaction can lead to self-heating. This is a known problem for larger batch sizes and can lead to temperatures exceeding the melting point of silicon (1414 °C). It was previously shown that the porous nature of the silicon products are related to the interconnectivity of the silicon nanocrystallites produced during MgTR.^7^ It is therefore expected that melting of the silicon crystallites will lead to a loss in porous structure.

**Fig. 1:**
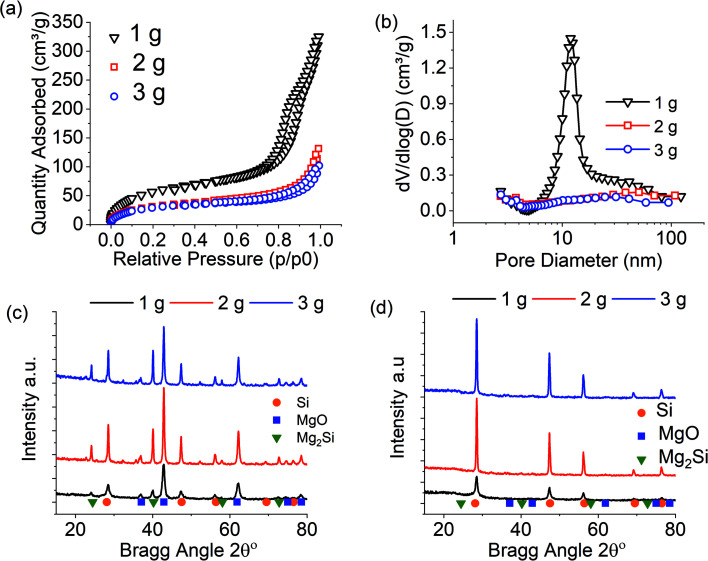
Characterisation of silicon produced during scale-up study of 1–3 g. MgTR conditions were: 2.5 : 1 Mg : SiO_2_ stoichiometry reacted for 6 hours at 650 °C. (a) N_2_ absorption isotherm, (b) BJH pore size distribution, (c) XRD of reaction products before washing with HCl, (d) XRD of reduction products after washing with HCl.

Fig. 1 presents the results for MgTR batch sizes of 1, 2 and 3 grams. For a full characterisation of silica precursors used, see reference.^7^ Note that for these reactions, optimised conditions, including a 2.5 : 1 Mg : SiO_2_ stoichiometric ratio, were used.^7^ In addition, slow ramping rates of 1 °C min^−1^ were used to prevent localised hotspots.^3^ The pore properties were greatly affected by the batch size in this study. When increasing the batch size above 1 g, a complete loss of mesoporosity was observed along with reduced surface area and pore volume (Fig. 1b and Table 1). The composition of the products before acid washing did not show any differences between the batches and all batches produced only silicon as a crystalline product upon washing (Fig. 1c and d). From Scherrer analysis of data in Fig. 1d, the silicon crystals displayed no nano-crystallites for 2 g and 3 g batch sizes, with crystal sizes being outside of Scherrer analysis range (>100 nm). Interestingly, the product purity was unaffected (remained within experimental errors) with the batch size (Table 1), thus confirming that the overall MgTR conversion was not influenced by the batch size, but self-heating had detrimental effects on the porosity and the microstructure of the products.

**Table 1:** Summary of sample properties obtained from the scale up study of 1–3 g without any thermal moderators. MgTR conditions were: 2.5 : 1 Mg : SiO_2_ stoichiometry reacted for 6 hours at 650 °C

Batch size (g) Mg + SiO_2_	SSA (m^2^ g^−1^)	BJH PV (cm^3^ g^−1^)	Purity wt% Si	Si_Cry_ size (nm)
1	230	0.48	57	10
2	112	0.18	50	>100
3	116	0.14	50	>100

**Fig. 2:**
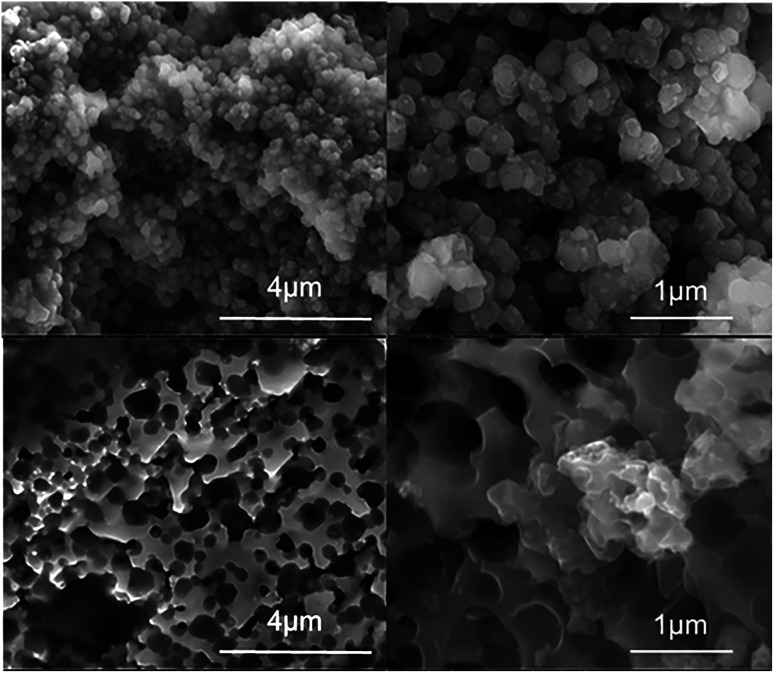
(a and b) Spherical morphology of a typical 1 g batch size reduction of BIS and (c and d) the melted ‘sponge like’ structure from a 2 g batch size reduction of BIS.

From SEM analysis (Fig. 2), there was also a clear change in the product morphology observed when going above 1 g batch sizes. The initial hierarchical “particulate” structure of the BIS template was retained for the 1 g batch. However, that structure was destroyed and replaced with macroscale melted particles for the larger batches (Fig. 2c and d), most likely fused together due to the formation of hotspots at larger batch sizes. It is apparent that for batch sizes above 1 g, self-heating occurs. The increase in silicon crystallite sizes to macroscale for larger batches suggests that the reduction temperature exceeded the melting point of silicon (1414 °C) even when the overall temperature in the furnace was maintained at 650 °C. The SEM images also suggest a complete change in morphology occurred from melting, and the porosity characterisation supported this view, consistent with the previously reported mechanism for pore formation.^7^

### 3.2 Sodium chloride as a thermal moderator

**Fig. 3:**
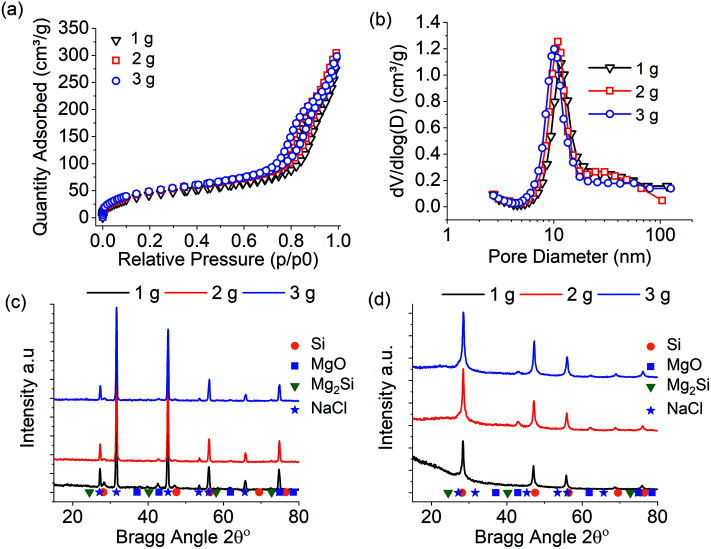
Characterisation of silicon produced during scale up study of 1–3 g, 2.5 : 1 Mg : SiO_2_ stoichiometry reacted for 6 hours at 650 °C, thermal moderator NaCl 1 : 5 ratio. (a) N_2_ absorption Isotherm, (b) BJH pore size distribution, (c) XRD of reaction products before washing with HCl, (d) XRD of reduction products after washing with HCl.

Previously, the self-heating of the MgTR reaction has been overcome by using unreactive thermal moderators such as sodium chloride.^3^ The above experiments for the scale up of BIS reduction to 3 g were repeated in the presence of NaCl thermal moderator. A mass ratio of 5 : 1 was used of reactants NaCl : (Mg + SiO_2_), while the effect of this ratio is investigated further below. The results are presented in Fig. 3. All samples display identical N_2_ isotherms and hence pore size distributions (Fig. 3 a, b and Table 2). The surface areas of the samples were typically lower than the 1 g reduction without thermal moderator (see Table 1), which is related to the increased purity of these samples – the lower amount of microporous BIS remaining leant to a decrease in surface area (discussed below). The pre-washing XRD of samples (Fig. 3c) were dominated by the strong diffraction signals from NaCl and hence masking MgTR products (MgO, Si, Mg_2_Si). However, after acid etching, it was clear that NaCl was completely removed, along with other by-products, to leave crystalline silicon (Fig. 3d). A small trace of MgO may be present in the 2 and 3 g batch size, perhaps enclosed within “closed” pores, although it is not believed to be significant. Scherrer analysis confirmed identical nanocrystallite sizes of ∼12 nm for each batch size.

**Table 2:** Summary of the properties of samples produced from the scale up study of 1–3 g when using NaCl as the thermal moderator. MgTR conditions were: 2.5 : 1 Mg : SiO_2_ stoichiometry reacted for 6 hours at 650 °C, thermal moderator NaCl 1 : 5 ratio

Batch size (g) Mg + SiO_2_	SSA (m^2^ g^−1^)	BJH PV (cm^3^ g^−1^)	wt% Si	Si_Cry_ size (nm)
1	162	0.43	65	12
2	182	0.46	71	12
3	184	0.45	62	11

The results presented in Fig. 3 support the view that NaCl acts as a thermal moderator in the MgTR to provide a nanocrystalline mesoporous silicon product. In addition, the purity of silicon product in the presence of NaCl (62–71 wt%) was greater than the equivalent purities when NaCl was not used (50–57 wt%) (Tables 1 and 2). A potential reason behind this increase in purity is believed to be related to the pestle and mortar mixing of the Mg, SiO_2_ and NaCl. The course nature of NaCl crystals can increase the homogeneity during the grinding step. The increased homogeneity of Mg and SiO_2_ may lead to less isolated SiO_2_ and hence increase the overall purity in the reduced sample. This also implies that a systematic future study of the effects of milling the precursors on the purity and properties of the silicon produced is warranted. In previous studies experiments have been performed where NaCl was deposited directly on the surface of reactions *via* the evaporation of a salt solution.^14,15^ This method may offer a future method of studying the mixing affects.

Initially, a large amount of NaCl was used in this study (1 : 5 (Mg + SiO_2_) : NaCl). Although NaCl is a cheap commodity, it is desirable to optimise the level of NaCl used. Additionally, it is important to understand the effect of NaCl ratio with reactants and its effect on silicon product properties which have previously been unexplored.

**Fig. 4:**
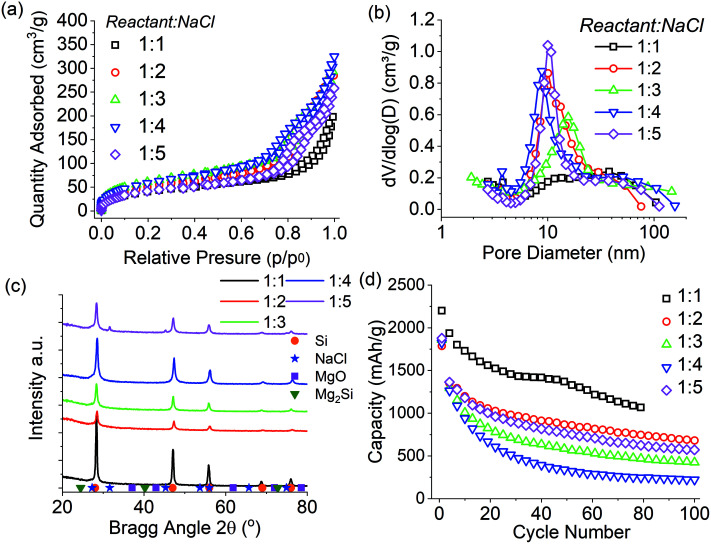
Characterisation of silicon produced during scale up study of 3 gram, 2.5 : 1 Mg : SiO_2_ stoichiometry reacted for 6 hours at 650 °C, thermal moderator NaCl ratio varied from 1 : 1–5. (a) N_2_ absorption Isotherm, (b) BJH pore size distribution, (c) XRD of reaction products after washing with HCl, (d) capacity and cycle life as anode in lithium half-cell.

The ratio of NaCl to reactant material (Mg + SiO_2_) was studied between 1 : 1 and 1 : 5 for a batch size of 3 g and the results are presented Fig. 4 and summarised in Table 3. Overall there was little difference in the pore size distributions when reducing the NaCl ratio from 1 : 5 to 1 : 2, except for the 1 : 3 ratio. All samples had similar mesopore distributions centred around 10 nm, while the sample prepared with 1 : 3 ratio had a slightly sifted distribution. The nanocrystallite size was 11–12 nm for all samples except that for the 1 : 1 sample. In contrast, the purity of samples of ratio 1 : 2–1 : 5 did vary between 56–73 wt% with no clear correlation; perhaps the grinding step was also affecting product purity as described above. For the reduction reaction performed with 1 : 1 reactant:NaCl ratio, there was an increase in the crystallite size to 28 nm and subsequent increase in pore size distribution, coupled with a reduction in pore volume, as has been previously reported.^7^ It is therefore concluded that reducing the NaCl ratio below 1 : 2 will inhibit the thermal moderation of the reduction reaction, leading to the crystallite size to rise and pore size distribution to broaden. However with a 1 : 1 the nano crystallinity of the sample was maintained and complete melting of the product structure was prevented.

**Table 3:** Summary of properties of samples obtained as a function of the NaCl ratio used. MgTR conditions were: 2.5 : 1 Mg : SiO_2_ stoichiometry reacted for 6 hours a 650 °C, thermal moderator NaCl ratio varied from 1 : 1–5

(SiO_2_+Mg) : NaCl ratio	SSA (m^2^ g^−1^)	BJH PV (cm^3^ g^−1^)	wt% Si	Si_Cry_ size (nm)
1 : 1	157	0.28	69	28
1 : 2	197	0.43	73	11
1 : 3	231	0.43	56	12
1 : 4	225	0.49	66	11
1 : 5	162	0.39	63	11

The lithium-ion battery performance of silicon produced in this study is presented in Fig. 4d. The silicon reduced at NaCl ratios of 1 : 2 to 1 : 5 showed similar cyclability with initial capacities between 1250–1900 mA h g^−1^ based upon silicon purity. These capacities reduced steadily with the 1 : 3, and the 1 : 4 ratio having the most significant capacity fade. The 1 : 1 ratio sample had the highest initial capacity of 2250 mA h g^−1^ but again shows similar capacity fade over 80 cycles. The increased capacity and cycling stability of the 1 : 1 sample is likely to be related to the increase in pore size distribution, which is known to decrease the stresses of volume expansion in silicon.^5^

**Fig. 5:**
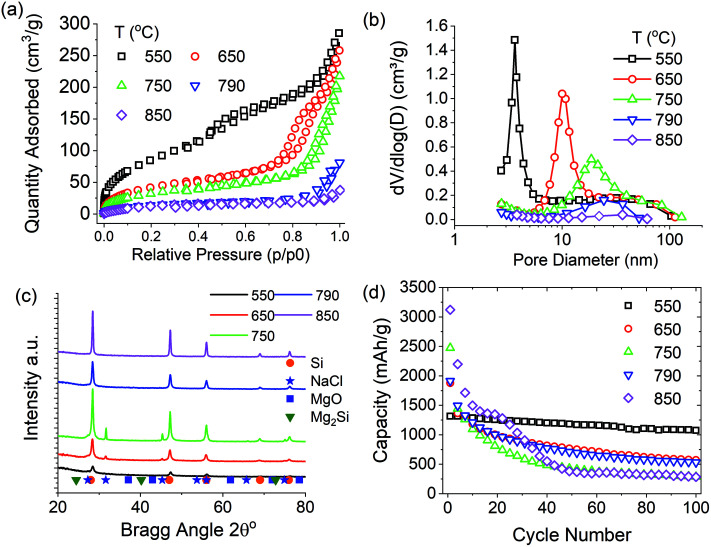
Characterisation of silicon produced during scale up study of 3 g, 2.5 : 1 Mg : SiO_2_ stoichiometry reacted for 6 hours between 550–850 °C, thermal moderator NaCl ratio 5 : 1. (a) N_2_ absorption Isotherms, (b) pore size distributions, (c) X-ray diffractograms and (d) electrode capacities over 100 cycles.

In previous work, we have shown that the upper temperature of MgTR significantly affects the pore properties, and hence the battery performance of product silicon.^7^ We have also shown above how NaCl can be used with BIS to maintain porous structures throughout reduction. A systematic study into the effect of NaCl ratio to reactants is lacking within the literature therefore this was further explored. Fig. 5 presents the results from MgTR with NaCl thermal moderator in a 1 : 5 ratio, reacted between 550 and 850 °C with reactants at a batch size of 3 g. As can be seen in Fig. 5a and b, the pore properties of the silicon product were strongly dependent on the reduction temperature. This is consistent with our previously proposed mechanism for pore formation driven by an increase in silicon crystallite size (see Table 4).^7^

**Table 4:** Summary of the properties of samples as a function of reduction temperature. MgTR conditions were: NaCl ratio 5 : 1, 2.5 : 1 Mg : SiO_2_ stoichiometry reacted for 6 hours temperatures between 550–850 °C

(SiO_2_+Mg) : NaCl ratio	Reduction temperature (°C)	SSA (m^2^ g^−1^)	BJH PV (cm^3^ g^−1^)	wt% Si	Si_Cry_ size (nm)
1 : 5	550	326	0.41	51	8
1 : 5	650	162	0.39	63	12
1 : 5	750	125	0.32	73	16
1 : 5	790	53	0.12	82	20
1 : 5	850	54	0.04	96	60

When considering the melting point of NaCl (801 °C), at reduction temperatures higher than 801 °C, molten NaCl will exist during the MgTR. After this phase transition, NaCl will not moderate the self-heating of the reaction. It is possible that molten NaCl may play some role in silicon formation, although this has not been studied in the literature and is beyond the scope of this study. However, it does explain why at 850 °C the NaCl no longer moderated the reduction and the mesoporosity was lost. A reduction reaction was performed at 790 °C to avoid NaCl melting and create a sample with high purity containing mesopores. This experiment was partially successful in producing high purity (82 wt%) mesoporous silicon, although the pore volume was low (0.12 cm^3^ g^−1^, Fig. 5b). Fig. 5c shows that NaCl was completely removed during acid washing, aside from the 750 °C sample where a small amount of magnesium oxide remained.

The performance of these silicon samples was evaluated as lithium-ion anode active materials (Fig. 5d). As expected, the high purity but low porosity sample reduced at 850 °C showed rapid capacity fade (97% after 100 cycles) due to lack of pore structure to absorb expansion stresses. The other mesoporous silicon samples obtained at 650, 750 and 790 °C all displayed similar capacity fade profiles: this suggests that their porous structure is not ideal for containing the stresses of volume expansion during cycling. This in turn is linked to the higher purity of these samples, as increasing silicon content increases the overall expansion during lithiation.

Previously, low purity silicon obtained at 550 °C showed excellent capacity retention (830 mA h g^−1^ initial capacity with 18% capacity fade over 100 cycles).^7^ In this study, the addition of NaCl to a 550 °C reduction has increased the purity from 29 to 51 wt%. As observed above, this is likely related to enhanced mixing when using NaCl. The high and stable capacity of this sample is displayed in Fig. 5d: the initial capacity of 1300 mA h g^−1^ faded by 19% over 100 cycles. The addition of NaCl has therefore increased the purity of these samples whilst simultaneously decreasing the reduction temperature, which is a key finding for making MgTR less energy intensive yet producing better anode materials.

## 4. Conclusion

The self-heating of MgTR is known to destroy templating structures. When scaling the synthesis of MgTR of BIS, the self-heating effect leads to loss of nano-crystallinity and porous structure. Here we investigated the effects of various parameters in the use of NaCl as a thermal moderator. In the presence of NaCl at ratios of 2–5 : 1, porous silicon structures could be produced at larger scales. Below 2 : 1 ratio, the NaCl was less able to moderate the self-heating effect. Further, owing to the melting point of NaCl (801 °C), it was unable to act as a successful thermal moderator for reduction temperatures above 800 °C.

The addition of NaCl in the grinding step of reactants is hypothesised to have led to an increase in yield for all reductions performed relative to their respective experiment without NaCl.^7^ This result meant that a high purity (52%) sample could be produced even at a low reduction temperature of 550 °C. This sample showed an initial capacity of 1300 mA h g^−1^ and excellent stability over 100 cycles. This sample shows a great promise for the MgTR as lowering the reduction temperature further increases the sustainability of this route. Our results have established pathways for scaling-up such that it can now be taken forward to target specific porous silicon properties. The results of this study suggest that low temperature reductions should be further explored. In addition, the mixing of NaCl with reactants has shown great effect on increasing product yield, this proposed mixing phenomenon needs further investigation.

## Conflicts of interest

There are no conflicts to declare.

## Supplementary Material
